# Analytical Insights into Ephaptic Coupling and Its Effect on Conduction Velocity

**DOI:** 10.1007/s00285-025-02315-9

**Published:** 2025-11-25

**Authors:** Ning Wei, Yoichiro Mori

**Affiliations:** 1 Department of Mathematics, Purdue University, 150 N. University St, West Lafayette, 47907 IN USA; 2 Department of Mathematics, University of Pennsylvania, 209 S 33rd St, Philadelphia, 19104 PA USA; 3 Department of Biology, University of Pennsylvania, 433 S University Ave, Philadelphia, 19104 PA USA

**Keywords:** Ephaptic coupling, Asymptotic theory, Conduction velocity, Action potential, 41A60, 92C30

## Abstract

Cardiovascular disease continues to be the leading cause of death in the United States. A major contributing factor is cardiac arrhythmia, which results from irregular electrical activity in the heart. On a tissue level, cardiac conduction involves the spread of action potentials (AP) across the heart, enabling coordinated contraction of the myocardium. On a cellular level, the transmission of signals between cells is facilitated by low-resistance pathways formed by gap junctions (GJs). Recent experimental studies have sparked discussion on whether GJs play a dominant role in cell communication. Interestingly, research has revealed that GJ knockout mice can still demonstrate signal propagation in the heart, albeit more slowly and discontinuously, indicating the presence of an alternative mechanism for cardiac conduction. Unlike GJ-mediated propagation, ephaptic coupling (EpC) has emerged as a distinct form of electrical transmission, characterized by contactless electrochemical signaling across the narrow intercalated discs (IDs) between cardiomyocytes. Advancements in cardiac research have highlighted the crucial role of EpC in restoring conduction by increasing conduction velocity (CV), reducing conduction block (CB), and terminating reentry arrhythmias, particularly when GJs are impaired. However, most EpC studies are either numerical or experimental, while analytical studies on ephaptic conduction–an equally important aspect of understanding EpC–remain extremely limited. In this paper, we applied asymptotic theory to calculate the CV in the presence of weak EpC. To achieve this, we developed both continuous and discrete models to describe ephaptic conduction along a strand of cells. Ionic dynamics were modeled using the piecewise linear and cubic functions. The resulting system represents a bistable system with weak EpC. We calculated an expression for CV in the presence of weak EpC for both models, and validated our analytical results with numerical simulations. Additionally, we showed that under weak EpC, CV can increase if the distribution of INa is more prominent on the end membrane.

## Introduction

The coordinated contraction of the heart depends on the orderly spread of the cardiac action potential (AP). This propagation occurs via gap junctions (GJs), which are low-resistance channels that directly link the cytoplasm of adjacent cardiac cells, allowing for the exchange of ions and small molecules. This network of connections enables the AP to activate neighboring cells in succession, generating a wave of depolarization across the myocardium. Traditionally, GJs have been recognized as the primary means of cellular communication (Beauchamp et al. 2004, 2012; Shaw and Rudy 1997; Vaidya et al. 2001).

However, ongoing theoretical and experimental research has raised concerns about the applicability of GJ-mediated microscale coupling to explain macroscopic propagation (Veeraraghavan et al. 2012; Gutstein et al. 2001; Danik et al. 2004; Yao et al. 2003). Specifically, pharmacologically uncoupling GJs using carbenoxolone alone does not lead to a noticeable slowdown in conduction (Veeraraghavan et al. 2012). Further doubts have emerged from studies involving GJ knockout mice (Gutstein et al. 2001; Danik et al. 2004; Yao et al. 2003). These mice are still able to maintain normal heart structure and function, despite the absence of detectable GJs. Taken together, these findings suggest that GJs may not be the only mechanism responsible for AP propagation in the heart.

Unlike GJ-mediated propagation, ephaptic coupling (EpC) has emerged as a unique form of electrical transmission, characterized by contactless electrochemical signaling across the narrow intercalated discs (ID) between cardiomyocytes (Hoshiko et al. 1959; Sperelakis and Mann 1977). Studies have supported the role of EpC as an alternative pathway for AP transmission when GJs are impaired (Sperelakis and Mann 1977; Sperelakis 2002; Pertsov and Medvinskiĭ 1976; Gutstein et al. 2001; Kucera et al. 2002; Mori et al. 2008; Wei et al. 2016; Rhett et al. 2012; Veeraraghavan et al. 2015; George et al. 2016; Greer-Short et al. 2017; Hichri et al. 2018; Raisch et al. 2018; Wei and Tolkacheva 2020; Veeraraghavan et al. 2018; Rhett et al. 2011). In particular, EpC has been demonstrated to enhance conduction velocity (CV) (Kucera et al. 2002; Wei et al. 2016; Lin and Keener 2010; Hand et al. 2009; Hand and Peskin 2010) and alleviate conduction block (CB) (Wei et al. 2016; Wei and Tolkacheva 2020) in the absence of functional GJs. Additionally, the authors demonstrated that EpC can terminate reentry in both healthy and ischemic hearts (Wei and Tolkacheva 2022). However, most EpC studies are either numerical (Kucera et al. 2002; Hand et al. 2009; Hand and Peskin 2010; Lin and Keener 2010, 2013; Mori et al. 2008; Wei and Tolkacheva 2020, 2022; Veeraraghavan et al. 2015; Wei et al. 2016; Tsumoto et al. 2020) or experimental (George et al. 2016, 2019; Hoeker et al. 2020; Veeraraghavan et al. 2015), aiming to uncover the physiological effects of EpC in the heart, primarily due to the lack of direct evidence supporting the existence of EpC. The sole analytical study on EpC (Copene and Keener 2008) investigates its feasibility in the absence of GJs. This study modeled two cells connected through a shared junctional cleft potential, with successful conduction being highly sensitive to specific parameters. The authors argued that the localization of fast sodium channels (INa) and a cleft-to-ground resistance that is neither too high nor too low are both essential for effective propagation. Nevertheless, the analytical expression for the CV of ephaptic conduction, which is equally crucial for understanding EpC, has not been explored in depth.

Applied analytical work on simplified models may complement biophysically realistic computational studies in gaining insight into EpC, especially given the complexity of this phenomenon. EpC in the absence of GJs is fundamentally discrete phenomenon at the cellular level as the voltage difference between two neighboring cells can be large. Nevertheless, approximate continuous models, because of their analytical tractability, may be useful in the study of EpC. Analysis of stability, oscillations, and bifurcations of continuous models may lead us to a better understanding of the impact of EpC on cardiac conduction and arrhythmogenesis. In this paper, we applied asymptotic theory to calculate the CV in the presence of weak EpC. To achieve this, we developed both continuous and discrete models to describe ephaptic conduction along a strand of cells. Ionic dynamics was modeled using the piecewise linear and cubic functions. The resulting system represents a bistable system with weak EpC. We calculated an expression for CV in the presence of weak EpC for both models, and validated our analytical results with numerical simulations. Additionally, we showed that under weak EpC, CV can increase if the distribution of INa is more prominent on the end membrane.

## Methods

To investigate cardiac propagation, we developed a mathematical model of ephaptic conduction along a strand of cells with the extracellular space grounded. In particular, each cell is treated as isopotential with an intracellular potential denoted by $V_i^m$ ($m = 1, 2, \dots , N$). The electrical potential in the cleft space, $V_c^{m - \frac{1}{2}}$ ($m = 2, 3, \dots , N$), differs from the extracellular potential, which is assumed to be constant in both space and time. For simplicity, we set $V_{\textrm{ext}} = 0$, so $V_i^m$ represents the transmembrane potential. The equations are formulated based on the current balance in both the intracellular and cleft spaces, as shown below.

Intracellular space ($$V_i^m$$, $$2 \le m\le N-1$$):

1 $$\begin{aligned}&A_{\textrm{end}}g_s (V_i^m-V_i^{m-1})+ A_{\textrm{end}}g_s (V_i^m-V_i^{m+1})+A_{\textrm{side}}\Big (C_m\frac{dV_i^m}{dt}+I_{\textrm{side}}(V_i^m)\Big ) \nonumber \\&+A_{\textrm{end}}\Big (C_m\frac{d(V_i^m-V_c^{m-\frac{1}{2}})}{dt}+I_{\textrm{end}}(V_i^m-V_c^{m-\frac{1}{2}})\Big )+A_{\textrm{end}}\Big (C_m\frac{d(V_i^m-V_c^{m+\frac{1}{2}})}{dt} \nonumber \\&+I_{\textrm{end}}(V_i^m-V_c^{m+\frac{1}{2}})\Big )=0, \end{aligned}$$

where $A_{\text {end}}=\pi r^2$ and $A_{\text {side}}=2\pi r L$ denote the cross-sectional and side areas of a cell, respectively; the values of *r* and *L* can be found in Hand and Peskin (2010); Wei et al. (2016); Copene and Keener (2008); $g_s$ denotes end-to-end GJ conductance per area (Wei et al. 2016; Hand and Peskin 2010; Copene and Keener 2008); $V_i^{m-1}$, $V_i^m$ and $V_i^{m+1}$ represent the intracellular potential of cells $m-1$, *m* and $m+1$, respectively. $V_c^{m-\frac{1}{2}}$ and $V_c^{m+\frac{1}{2}}$ denote the electric potential in the cleft spaces at locations $m-\frac{1}{2}$ and $m+\frac{1}{2}$, respectively. $C_m$ is the membrane capacitance per area. $I_\text {end}$ and $I_\text {side}$ represent the outward ionic current per area of the side and end membranes, respectively. Note that for $m=1$, the terms $A_{\textrm{end}}g_s (V_i^m-V_i^{m-1})$ and $A_{\textrm{end}}\Big (C_m\frac{d(V_i^m-V_c^{m-\frac{1}{2}})}{dt}+I_{\textrm{end}}(V_i^m-V_c^{m-\frac{1}{2}})\Big )$ are omitted from Eq. 1. Similarly, for $m=N$, the terms $A_{\textrm{end}}g_s (V_i^m-V_i^{m+1})$ and $A_{\textrm{end}}\Big (C_m\frac{d(V_i^m-V_c^{m+\frac{1}{2}})}{dt}+I_{\textrm{end}}(V_i^m-V_c^{m+\frac{1}{2}})\Big )$ are omitted from Eq. 1.

Cleft space ($$V_c^{m-\frac{1}{2}}$$, $$2 \le m\le N$$):

2 $$\begin{aligned} \begin{aligned}&-A_{\textrm{end}}\Big (C_m\frac{d(V_i^{m-1}-V_c^{m-\frac{1}{2}})}{dt}+I_{\textrm{end}}(V_i^{m-1}-V_c^{m-\frac{1}{2}})\Big )-A_{\textrm{end}}\Big (C_m\frac{d(V_i^m-V_c^{m-\frac{1}{2}})}{dt}\\&+I_{\textrm{end}}(V_i^m-V_c^{m-\frac{1}{2}})\Big )+\frac{V_c^{m-\frac{1}{2}}}{R_c}=0, \end{aligned} \end{aligned}$$

where $R_c$ denotes the cleft-to-ground resistance, which is inversely proportional to the cleft width ($d_{\textrm{cleft}}$) using formulas from Hand and Peskin (2010). The value of $d_{\textrm{cleft}}$ is not biologically measurable with current experimental techniques; however, a range of 2–115 nm has been reported (Hand and Peskin 2010). Large $R_c$ (small $d_{\textrm{cleft}}$) values indicate strong EpC, while small $R_c$ (large $d_{\textrm{cleft}}$) values indicate weak EpC.

It is well-known that detailed physiological models, such as the Luo-Rudy dynamic model or the Hodgkin-Huxley model, incorporate complex ionic current and gating dynamics, which make mathematical analysis challenging. Drawing inspiration from the two-current model proposed in Mitchell and Schaeffer (2003); Copene and Keener (2008), we simulated the ionic current flow in our model using a modified version of the two-current model for cardiac action potentials. Since we are primarily concerned with computing the CV, which is directly related to the upstroke of the action potential, we simplified the model by neglecting gating variables and recovery dynamics. As a result, we modeled the membrane dynamics with an inward $\text {Na}^+$ current, gated by a single variable, and an outward $\text {K}^+$ current, which captures the essential features of an action potential (Copene and Keener 2008). The following equation is used to model cell excitation:

$$\begin{aligned} I(V)=g_{\textrm{Na}}m_\infty (V)(V-V_{\textrm{Na}})+g_{\textrm{K}}(V-V_{\textrm{K}}),\end{aligned}$$

where $g_{\textrm{Na}}$ and $g_{\textrm{K}}$ are conductances per unit area for $\textrm{Na}^+$ and $\textrm{K}^+$ current, respectively; $V_{\textrm{Na}}$ and $V_{\textrm{K}}$ are equilibrium potentials for $\textrm{Na}^+$ and $\textrm{K}^+$ current, respectively. For simplicity, we take $m_\infty (V)=H(V-V_\text {th})$, where *H* is the Heaviside step function and $V_\text {th}$ is the threshold potential for excitation.

The resulting system of equations can be nondimensionalized as follows:

3 $$\begin{aligned} V_i^m=\phi _i^mV^*+V_{\textrm{K}} (1 \le m\le N), \end{aligned}$$

4 $$\begin{aligned} V_c^{m-\frac{1}{2}}=\phi _c^{m-\frac{1}{2}}V^*(1 \le m\le N), \end{aligned}$$

where $V^*=V_{\textrm{Na}}-V_{\textrm{K}}$. Therefore, the nondimensional potentials range between 0 and 1 (Copene and Keener 2008). Additionally, time is rescaled as $t=t^*\tau$. The nondimensional system of equations can be written as follows:

5 $$\begin{aligned} \begin{aligned}&\frac{d \phi _i^m}{d \tau }+\alpha \frac{d( \phi _i^m-\phi _c^{m-\frac{1}{2}})}{d \tau }+ \alpha \frac{d (\phi _i^m-\phi _c^{m+\frac{1}{2}})}{d \tau }+ \gamma _s(\phi _i^m-\phi _i^{m-1}) + \gamma _s(\phi _i^m-\phi _i^{m+1})\\&=f_{\textrm{side}}(\phi _i^m)+\alpha f_{\textrm{end}}(\phi _i^m-\phi _c^{m-\frac{1}{2}})+\alpha f_{\textrm{end}}(\phi _i^m-\phi _c^{m+\frac{1}{2}}), \end{aligned} \end{aligned}$$

where $\alpha =\frac{A_{\textrm{end}}}{A_{\textrm{side}}}$ and $\gamma _s=\frac{\alpha g_s t^*}{C_m }$ are dimensionless parameters representing the ratio of the end membrane area to the side membrane area and the end-to-end GJs, respectively. $f_{\textrm{side}}$ and $f_{\textrm{end}}$ are bistable functions that represent the nondimensionalized ionic currents across the side and end membranes, respectively.

6 $$\begin{aligned} \begin{aligned}&\alpha \frac{d(\phi _i^{m-1}-\phi _c^{m-\frac{1}{2}})}{d\tau }+ \alpha \frac{d(\phi _i^m-\phi _c^{m-\frac{1}{2}})}{d\tau }=\alpha f_{\textrm{end}}(\phi _i^{m-1}-\phi _c^{m-\frac{1}{2}})\\&+\alpha f_{\textrm{end}}(\phi _i^m-\phi _c^{m-\frac{1}{2}})+\frac{1}{\delta } \phi _c^{m-\frac{1}{2}}, \end{aligned} \end{aligned}$$

where $\delta =\frac{R_cA_{\textrm{side}}C_m}{t^*}$ is a dimensionless parameter representing the cleft-to-ground resistance. Note that

7 $$\begin{aligned} \begin{aligned}&f_{\textrm{side}}(\phi )=-\gamma _{\textrm{Na}}m_\infty ^{\textrm{side}}(\phi )(\phi -\phi _{\textrm{Na}})-\gamma _{\textrm{K}}(\phi -\phi _{\textrm{K}}),\\&f_{\textrm{end}}(\phi )=-\beta \gamma _{\textrm{Na}}m_\infty ^{\textrm{end}}(\phi )(\phi -\phi _{\textrm{Na}})-\gamma _{\textrm{K}}(\phi -\phi _{\textrm{K}}), \end{aligned} \end{aligned}$$

where $\gamma _{\textrm{Na}}=\frac{g_{\textrm{Na}}t^*}{C_m}$ and $\gamma _{\textrm{K}}=\frac{g_{\textrm{K}}t^*}{C_m}$ are the nondimensional conductances for $\mathrm Na^+$ and $\mathrm K^+$ conductance, respectively. The nondimensional equilibrium potentials $\phi _{\textrm{Na}}$ and $\phi _{\textrm{K}}$ are set to 1 and 0, respectively. Additionally, $m_\infty ^{\textrm{side}}(\phi )=H(\phi -\phi _{\textrm{th}})$, $m_\infty ^{\textrm{end}}(\phi )=H(\phi -\frac{\phi _{\textrm{th}}}{\beta })$, where *H* is the Heaviside step function and $\beta$ is a parameter that quantifies the localization of INa channels to the cleft (Rhett et al. 2012). In particular, $\beta = 1$ corresponds to uniformly distributed $I_{\textrm{Na}}$ channels, whereas $\beta > 1$ indicates localization, with values reported in Hand and Peskin (2010). A $\beta$ greater than 1 results in increased $I_{\textrm{Na}}$ conductance and a lower threshold for cell excitation at the end membrane. In addition to the piecewise linear ionic model, we also examined the cubic ionic model described below:

8 $$\begin{aligned} \begin{aligned} f_{\textrm{side}}(\phi )=-A^2\phi (\phi -\epsilon _{\textrm{side}})(\phi -1),\\ f_{\textrm{end}}(\phi )=-A^2\beta \phi (\phi -\epsilon _{\textrm{end}})(\phi -1),\\ \end{aligned} \end{aligned}$$

where $\epsilon _{\textrm{end}}=\frac{\epsilon _{\textrm{side}}}{\beta }$. Similarly to the piecewise-linear ionic model, $\beta >1$ results in larger $I_{\textrm{Na}}$ and a lower excitation threshold at the end membrane. The parameter values $\beta$, $\gamma _{\textrm{Na}}$, $\gamma _{\textrm{K}}$, $\sigma$, $\phi _{\textrm{th}}$, and *A* are taken from Copene and Keener (2008).

## Results

The goal of this study is to derive an expression for the CV under the assumption of weak EpC (small $\alpha$) using asymptotic analysis. This weak-EpC regime is of interest because conventional models ignore EpC and thus correspond to the $\alpha \rightarrow 0$ limit.

### Conduction speed of continuous model when $\alpha$ is small

#### Piecewise linear ionic model

It is important to note that when $\alpha$ is small (for example as $\alpha \rightarrow 0$), $\phi _c\rightarrow$ 0 according to Eq. (16), which reduces the system to the classical monodomain model. Therefore, small values of $\alpha$ serve as a measure of weak EpC. We begin by deriving the continuous model from the discrete model. The left-hand side of equation (5) leads to the following expression:

9 $$\begin{aligned} &  \frac{d \phi _i^m}{d \tau }+\alpha \frac{d( \phi _i^m-\phi _c^{m-\frac{1}{2}})}{d \tau }+\alpha \frac{d (\phi _i^m-\phi _c^{m+\frac{1}{2}})}{d \tau }+\gamma _s(2\phi _i^m-\phi _i^{m-1}-\phi _i^{m+1}) \nonumber \\ &  \quad =\frac{d \phi _i^m}{d \tau }+\alpha \frac{d( 2\phi _i^m-\phi _c^{m-\frac{1}{2}}-\phi _c^{m+\frac{1}{2}})}{d\tau }+\gamma _s\big (2\phi _i^m-(\phi _i^m-\bigtriangleup x \frac{\partial \phi _i^m}{\partial x}+\frac{\bigtriangleup x^2}{2}\frac{\partial ^2\phi _i^m}{\partial x^2})\nonumber \\ &  \qquad -(\phi _i^m+\bigtriangleup x\frac{\partial \phi _i^m}{\partial x}+\frac{\bigtriangleup x^2}{2}\frac{\partial ^2\phi _i^m}{\partial x^2})\big )\nonumber \\ &  =\frac{d \phi _i^m}{d \tau }+\alpha \frac{d\big ( 2\phi _i^m-(\phi _c^{m}-\frac{\bigtriangleup x}{2}\frac{\partial \phi _c^m}{\partial x}+\frac{\bigtriangleup x^2}{8}\frac{\partial ^2 \phi _c^m}{\partial x^2})-(\phi _c^{m}+\frac{\bigtriangleup x}{2}\frac{\partial \phi _c^m}{\partial x}+\frac{\bigtriangleup x^2}{8}\frac{\partial ^2 \phi _c^m}{\partial x^2})\big )}{d\tau }\nonumber \\ &  \quad +\gamma _s(-\bigtriangleup x^2 \frac{\partial ^2\phi _i^m}{\partial x^2})\nonumber \\ &  =\frac{d \phi _i^m}{d \tau }+2\alpha \frac{d(\phi _i^m-\phi _c^m-\frac{\bigtriangleup x^2}{8}\frac{\partial ^2 \phi _c^m}{\partial x^2})}{d\tau }-\gamma _s\bigtriangleup x^2 \frac{\partial ^2\phi _i^m}{\partial x^2}. \end{aligned}$$

Additionally, the right-hand side of Eq. (5) leads to the following expression:

10 $$\begin{aligned} \begin{aligned}&f_{\textrm{side}}(\phi _i^m)+\alpha f_{\textrm{end}}(\phi _i^m-\phi _c^{m-\frac{1}{2}})+\alpha f_{\textrm{end}}(\phi _i^m-\phi _c^{m+\frac{1}{2}}) \\&\quad =f_{\textrm{side}}(\phi _i^m)+\alpha \big (f_{\textrm{end}}(\phi _i^m-\phi _c^m)+f'_{\textrm{end}}(\phi _i^m-\phi _c^m)(\phi _c^m-\phi _c^{m-\frac{1}{2}})\big )\\&\quad \quad +\alpha \big (f_{\textrm{end}}(\phi _i^m-\phi _c^m)+f'_{\textrm{end}}(\phi _i^m-\phi _c^m)(\phi _c^m-\phi _c^{m+\frac{1}{2}})\big )\\&\quad =f_{\textrm{side}}(\phi _i^m)+2\alpha f_{\textrm{end}}(\phi _i^m-\phi _c^m)+\alpha f'_{\textrm{end}}(\phi _i^m-\phi _c^m)(2\phi _c^m-\phi _c^{m-\frac{1}{2}}-\phi _c^{m+\frac{1}{2}})\\&\quad = f_{\textrm{side}}(\phi _i^m)+2\alpha f_{\textrm{end}}(\phi _i^m-\phi _c^m)+\alpha f'_{\textrm{end}}(\phi _i^m-\phi _c^m)\big (2\phi _c^m-(\phi _c^m-\frac{\bigtriangleup x}{2}\frac{\partial \phi _c^m}{\partial x}\\&\qquad +\frac{\bigtriangleup x^2}{8}\frac{\partial ^2 \phi _c^m}{\partial x^2})- (\phi _c^m+\frac{\bigtriangleup x}{2}\frac{\partial \phi _c^m}{\partial x}+\frac{\bigtriangleup x^2}{8}\frac{\partial ^2 \phi _c^m}{\partial x^2})\big )\\&\quad =f_{\textrm{side}}(\phi _i^m)+2\alpha f_{\textrm{end}}(\phi _i^m-\phi _c^m)-\alpha f'_{\textrm{end}}(\phi _i^m-\phi _c^m)\frac{\bigtriangleup x^2}{4}\frac{\partial ^2 \phi _c^m}{\partial x^2}. \end{aligned} \end{aligned}$$

To retain the diffusion term, we set $\gamma _s\bigtriangleup x^2=\gamma ^*$ as a constant. Therefore, we assume $\bigtriangleup x=\alpha$ and $\gamma _s=\frac{\gamma ^*}{\alpha ^2}$. By keeping O(1) and O($\alpha$) terms, Eq. (5) can be rewritten as follows:

11 $$\begin{aligned} \begin{aligned} \frac{d \phi _i^m}{d \tau }+2\alpha \frac{d(\phi _i^m-\phi _c^m)}{d\tau }=\gamma ^* \frac{\partial ^2\phi _i^m}{\partial x^2}+f_{\textrm{side}}(\phi _i^m)+2\alpha f_{\textrm{end}}(\phi _i^m-\phi _c^m). \end{aligned} \end{aligned}$$

Similarly, the left-hand side of Eq. (6) can be expressed as the following:

12 $$\begin{aligned} &  \alpha \frac{d(\phi _{i}^{m-1}+\phi _i^m-2\phi _c^{m-\frac{1}{2}})}{d\tau } \nonumber \\ &  \quad =\alpha \frac{d(2\phi _{i}^{m-\frac{1}{2}}-\frac{\bigtriangleup x}{2}\frac{\partial \phi _i^{m-\frac{1}{2}}}{\partial x}+\frac{\bigtriangleup x^2}{8}\frac{\partial ^2\phi _i^{m-\frac{1}{2}}}{\partial x^2}+\frac{\bigtriangleup x}{2}\frac{\partial \phi _i^{m-\frac{1}{2}}}{\partial x}+\frac{\bigtriangleup x^2}{8}\frac{\partial ^2\phi _i^{m-\frac{1}{2}}}{\partial x^2}-2\phi _c^{m-\frac{1}{2}})}{d\tau } \nonumber \\ &  =2\alpha \frac{d(\phi _i^{m-\frac{1}{2}}+\frac{\bigtriangleup x^2}{8}\frac{\partial ^2\phi _i^{m-\frac{1}{2}}}{\partial x^2}-\phi _c^{m-\frac{1}{2}})}{d\tau }. \end{aligned}$$

Moreover, the right-hand side of Eq. (6) can be expressed as follows:

13 $$\begin{aligned} &  \alpha f_{\textrm{end}}(\phi _i^{m-1}-\phi _c^{m-\frac{1}{2}})+\alpha f_{\textrm{end}}(\phi _i^m-\phi _c^{m-\frac{1}{2}})+\frac{1}{\delta }\phi _c^{m-\frac{1}{2}}\nonumber \\ &  =\alpha f_{\textrm{end}}(\phi _i^{m-\frac{1}{2}}-\phi _c^{m-\frac{1}{2}})+\alpha f'_{\textrm{end}}(\phi _i^{m-\frac{1}{2}}-\phi _c^{m-\frac{1}{2}})(\phi _i^{m-1}-\phi _i^{m-\frac{1}{2}})\nonumber \\ &  +\alpha f_{\textrm{end}}(\phi _i^{m-\frac{1}{2}}-\phi _c^{m-\frac{1}{2}})+\alpha f'_{\textrm{end}}(\phi _i^{m-\frac{1}{2}}-\phi _c^{m-\frac{1}{2}})(\phi _i^m-\phi _i^{m-\frac{1}{2}})+\frac{1}{\delta }\phi _c^{m-\frac{1}{2}}\nonumber \\ &  =2\alpha f_{\textrm{end}}(\phi _i^{m-\frac{1}{2}}-\phi _c^{m-\frac{1}{2}})+\alpha f'_{\textrm{end}}(\phi _i^{m-\frac{1}{2}}-\phi _c^{m-\frac{1}{2}})(\phi _i^{m-1}+\phi _i^m-2\phi _i^{m-\frac{1}{2}})+\frac{1}{\delta }\phi _c^{m-\frac{1}{2}}\nonumber \\ &  =2\alpha f_{\textrm{end}}(\phi _i^{m-\frac{1}{2}}-\phi _c^{m-\frac{1}{2}})+\alpha f'_{\textrm{end}}(\phi _i^{m-\frac{1}{2}}-\phi _c^{m-\frac{1}{2}})\big (\phi _i^{m-\frac{1}{2}}-\frac{\bigtriangleup x}{2}\frac{\partial \phi _i^{m-\frac{1}{2}}}{\partial x}\nonumber \\ &  +\frac{\bigtriangleup x^2}{8}\frac{\partial ^2 \phi _i^{m-\frac{1}{2}}}{\partial x^2}+\phi _i^{m-\frac{1}{2}}+\frac{\bigtriangleup x}{2}\frac{\partial \phi _i^{m-\frac{1}{2}}}{\partial x}+\frac{\bigtriangleup x^2}{8}\frac{\partial ^2 \phi _i^{m-\frac{1}{2}}}{\partial x^2}-2\phi _i^{m-\frac{1}{2}}\big )+\frac{1}{\delta }\phi _c^{m-\frac{1}{2}} \nonumber \\ &  =2\alpha f_{\textrm{end}}(\phi _i^{m-\frac{1}{2}}-\phi _c^{m-\frac{1}{2}})+\alpha f'_{\textrm{end}}(\phi _i^{m-\frac{1}{2}}-\phi _c^{m-\frac{1}{2}})\frac{\bigtriangleup x^2}{4}\frac{\partial ^2 \phi _i^{m-\frac{1}{2}}}{\partial x^2}+\frac{1}{\delta }\phi _c^{m-\frac{1}{2}}. \end{aligned}$$

By retaining the O(1) and O($\alpha$) terms, Eq. (6) can be expressed as follows:

14 $$\begin{aligned} 2\alpha \frac{d(\phi _i^{m-\frac{1}{2}}-\phi _c^{m-\frac{1}{2}})}{d\tau }= 2\alpha f_{\textrm{end}}(\phi _i^{m-\frac{1}{2}}-\phi _c^{m-\frac{1}{2}})+\frac{1}{\delta }\phi _c^{m-\frac{1}{2}}. \end{aligned}$$

Without loss of generality, we assumed $\gamma ^*=1$. After removing the indices and combining the two equations above, we arrived at the following dimensionless continuous model.

15 $$\begin{aligned} \frac{\partial \phi _i}{\partial \tau }=\frac{\partial ^2\phi _i}{\partial x^2}+f_{\textrm{side}}( \phi _i)-\sigma \phi _c, \end{aligned}$$

16 $$\begin{aligned} \frac{\partial (\phi _i-\phi _c)}{\partial \tau }=f_{\textrm{end}}(\phi _i-\phi _c)+\frac{\sigma }{2\alpha }\phi _c, \end{aligned}$$

where $\sigma =\frac{1}{\delta }$ denotes the nondimensional cleft-to-ground conductance.

We considered a solution of the form $\phi _i(\tau ,x)=\Phi _i(x+c\tau )=\Phi _i(\xi )$, $\phi _c(\tau ,x)=\Phi _c(x+c\tau )=\Phi _c(\xi )$. Here *c* represents the CV of the traveling front and $\xi$ is the traveling wave coordinate. The Eqs. (15) - (16) then become:

17 $$\begin{aligned} \Phi _i''-c\Phi _i'+f_{\textrm{side}}(\Phi _i)=\sigma \Phi _c, \end{aligned}$$

18 $$\begin{aligned} c(\Phi _i'-\Phi _c') =f_{\textrm{end}}(\Phi _i-\Phi _c)+ \frac{\sigma }{2\alpha }\Phi _c, \end{aligned}$$

where $\Phi _i$ provides a transition between two rest points. Therefore, we can express the previously mentioned system as a set of three first-order differential equations, as follows:

19 $$\begin{aligned} \begin{aligned} \Phi _i'&=\Psi _i,\\ \Psi _i'-c\Psi _i+f_{\textrm{side}}(\Phi _i)&=\sigma \Phi _c,\\ c(\Psi _i-\Phi _c')&=f_{\textrm{end}}(\Phi _i-\Phi _c)+\frac{\sigma }{2\alpha }\Phi _c. \end{aligned} \end{aligned}$$

To find traveling front solutions for the bistable equation, we sought a solution to Eq. (19) that connects the rest points $P_1$ and $P_2$ in the form $$\begin{bmatrix} \Phi _i \\ \Phi _c \\ \Psi _i \end{bmatrix}$$. Specifically, we have

$$\begin{aligned}P_1=(0, 0, 0),\;\textrm{and}\; P_2=\Big (\frac{\gamma _{\textrm{Na}}-\sigma \Phi _c^*}{\gamma _{\textrm{Na}}+\gamma _{\textrm{K}}},\Phi _c^*, 0\Big ),\end{aligned}$$

where

$$\begin{aligned}\Phi _c^*=\frac{1}{\beta \gamma _{\textrm{Na}}+\gamma _{\textrm{K}}+\frac{\sigma }{2\alpha }+\frac{\sigma ( \beta \gamma _{\textrm{Na}}+\gamma _{\textrm{K}})}{\gamma _{\textrm{Na}}+\gamma _{\textrm{K}}}}\frac{(1-\beta )\gamma _{\textrm{Na}}\gamma _{\textrm{K}}}{\gamma _{\textrm{Na}}+\gamma _{\textrm{K}}}\le 0\; (\beta \ge 1).\end{aligned}$$

Note that $\Phi _c^*=0$ at $\beta =1$; $\Phi _c^*<0$ for $\beta >1$.

We linearized Eq. (19) around the two rest points as follows:

$$\begin{aligned}\frac{d}{d\tau }\begin{bmatrix} \Phi _i \\ \Phi _c \\ \Psi _i \end{bmatrix} = J_{\textrm{ss}} \begin{bmatrix} \Phi _i \\ \Phi _c \\ \Psi _i \end{bmatrix},\end{aligned}$$

where $J_{\textrm{ss}}$ is the Jacobin matrix evaluated at the rest points. At point $P_1$, the Jacobin matrix is

$$\begin{aligned}J_{\textrm{ss}}=\begin{bmatrix} 0 & 0 & 1 \\ \frac{\gamma _{\textrm{K}}}{c} & -\frac{\gamma _{\textrm{K}}}{c}-\frac{\sigma }{2\alpha c} & 1 \\ \gamma _{\textrm{K}} & \sigma & c \end{bmatrix}. \end{aligned}$$

The characteristic equation is

$$\begin{aligned}-\mu ^3+(c-\frac{\gamma _{\textrm{K}}}{c}-\frac{\sigma }{2\alpha c})\mu ^2+(2\gamma _K+\frac{\sigma }{2\alpha }+\sigma )\mu +(\frac{\gamma _{\textrm{K}}\sigma }{c}+\frac{\gamma _{\textrm{K}}^2}{c}+\frac{\gamma _{\textrm{K}}\sigma }{2\alpha c})=0.\end{aligned}$$

Therefore, we have

$$\begin{aligned}\mu _1\mu _2\mu _3=\frac{\gamma _{\textrm{K}}\sigma }{c}+\frac{\gamma _{\textrm{K}}^2}{c}+\frac{\gamma _{\textrm{K}}\sigma }{2\alpha c}>0,\\\mu _1+\mu _2+\mu _3=c-\frac{\gamma _{\textrm{K}}}{c}-\frac{\sigma }{2\alpha c},\\\mu _1\mu _2+\mu _1\mu _3+\mu _2\mu _3=-(2\gamma _K+\frac{\sigma }{2\alpha }+\sigma )<0.\end{aligned}$$

From these results, we can conclude that two eigenvalues (or the real parts of two complex eigenvalues) are negative, while the other is positive. Similarly, at the second rest point $P_2$, the Jacobin matrix is

$$\begin{aligned}J_{\textrm{ss}}=\begin{bmatrix} 0 & 0 & 1 \\ \frac{\beta \gamma _{\textrm{Na}}+\gamma _{\textrm{K}}}{c} & - \frac{\beta \gamma _{\textrm{Na}}+\gamma _{\textrm{K}}}{c}-\frac{\sigma }{2\alpha c} & 1 \\ \gamma _{\textrm{Na}}+\gamma _{\textrm{K}} & \sigma & c \end{bmatrix}. \end{aligned}$$

The characteristic equation is

$$\begin{aligned}-\mu ^3+(c-\frac{\beta \gamma _{\textrm{Na}}+\gamma _{\textrm{K}}}{c}-\frac{\sigma }{2\alpha c})\mu ^2+(\beta \gamma _{\textrm{Na}}+\gamma _K+\gamma _{\textrm{Na}}+\gamma _K+\frac{\sigma }{2\alpha }+\sigma )\mu \\+\Big (\frac{\sigma }{c}(\beta \gamma _{\textrm{Na}}+\gamma _{\textrm{K}})+\frac{(\gamma _{\textrm{Na}}+\gamma _{\textrm{K}})(\beta \gamma _{\textrm{Na}}+\gamma _{\textrm{K}})}{c}+\frac{\sigma }{2\alpha c}(\gamma _{\textrm{Na}}+\gamma _{\textrm{K}})\Big )=0.\end{aligned}$$

Thus, we have

$$\begin{aligned}\mu _1\mu _2\mu _3=\frac{\sigma }{c}(\beta \gamma _{\textrm{Na}}+\gamma _{\textrm{K}})+\frac{(\gamma _{\textrm{Na}}+\gamma _{\textrm{K}})(\beta \gamma _{\textrm{Na}}+\gamma _{\textrm{K}})}{c}+\frac{\sigma }{2\alpha c}(\gamma _{\textrm{Na}}+\gamma _{\textrm{K}})>0\\\mu _1+\mu _2+\mu _3=c-\frac{\beta \gamma _{\textrm{Na}}+\gamma _{\textrm{K}}}{c}-\frac{\sigma }{2\alpha c}\\\mu _1\mu _2+\mu _1\mu _3+\mu _2\mu _3=-(\beta \gamma _{\textrm{Na}}+\gamma _K+\gamma _{\textrm{Na}}+\gamma _K+\frac{\sigma }{2\alpha }+\sigma )<0.\end{aligned}$$

Again, we observed that two eigenvalues (or the real parts of two complex conjugate eigenvalues) are negative, while the other is positive. Since Eq. (19) has one free variable *c*, and considering the signs of the eigenvalues at the rest points, we concluded that there exists a heteroclinic trajectory connecting the two rest points.

The objective of our study is to select the parameter $c$ such that the trajectory originating from $P_1$ at $\xi = -\infty$ connects with $P_2$ at $\xi = \infty$. As $\alpha \rightarrow 0$, $\Phi _c$ tends to zero. Consequently, Eqs. (17) and (18) reduce to the standard monodomain model. In the monodomain model, explicit expressions for CV and potential are well-known. Thus, we can derive the following perturbation argument for CV and potentials around $\alpha$:

$$\begin{aligned}c=c_0+c_1\alpha +\cdots \\\Phi _i=\Phi _{i0}+\Phi _{i1}\alpha +\cdots \\\Phi _c=\Phi _{c0}+\Phi _{c1}\alpha +\cdots \end{aligned}$$

Substituting into Eqs. (17) and (18) and we obtain the following equations:

20 $$\begin{aligned} \begin{aligned}&(\Phi _{i0}''+\Phi _{i1}''\alpha +\cdots )-(c_0+c_1\alpha +\cdots )(\Phi _{i0}'+\Phi _{i1}'\alpha +\cdots )+f_{\textrm{side}}(\Phi _{i0}+\Phi _{i1}\alpha +\cdots )\\&=\sigma (\Phi _{c0}+\Phi _{c1}\alpha +\cdots ) \end{aligned} \end{aligned}$$

21 $$\begin{aligned} \begin{aligned}&2\alpha (c_0+c_1\alpha +\cdots )(\Phi _{i0}'+\Phi _{i1}'\alpha +\cdots -\Phi _{c0}'-\Phi _{c1}'\alpha -\cdots )\\&=2\alpha f_{\textrm{end}}(\Phi _{i0}+\Phi _{i1}\alpha +\cdots -\Phi _{c0}-\Phi _{c1}\alpha -\cdots )+\sigma (\Phi _{c0}+\Phi _{c1}\alpha +\cdots ) \end{aligned} \end{aligned}$$

We reordered the terms in powers of $\alpha$, yielding the following expressions:

22 $$\begin{aligned} \begin{aligned}&\Big (\Phi _{i0}''-c_0\Phi _{i0}'+f_{\textrm{side}}(\Phi _{i0})-\sigma \Phi _{c0}\Big )+\alpha \Big ( \Phi _{i1}''-c_0\Phi _{i1}'-c_1\Phi _{i0}'+f_{\textrm{side}}'(\Phi _{i0})\Phi _{i1}-\sigma \Phi _{c1}\Big )\\&+\cdots =0 \end{aligned} \end{aligned}$$

23 $$\begin{aligned} -\sigma \Phi _{c0}+\alpha \Big (2c_0(\Phi _{i0}-\Phi _{c0})'-2f_{\textrm{end}}(\Phi _{i0}-\Phi _{c0})-\sigma \Phi _{c1} \Big )+\cdots =0 \end{aligned}$$

Thus, the leading terms are given by:

24 $$\begin{aligned} \begin{aligned}&\Phi _{i0}''-c_0\Phi _{i0}'+f_{\textrm{side}}(\Phi _{i0})-\sigma \Phi _{c0}=0, \\&-\sigma \Phi _{c0}=0 \end{aligned} \end{aligned}$$

From Eq. (24), we have $\Phi _{c0}=0$. Eq. (24) simplifies to $\Phi _{i0}''-c_0\Phi _{i0}'+f_{\textrm{side}}(\Phi _{i0})=0$, which is the classical monodomain equation. To compute $\Phi _{i0}$, the leading term of $\Phi _i$, we considered the case where $f$ is a piecewise linear function. For $\Phi _{i0} < \Phi _{\textrm{th}}$, Eq. (24) becomes:

25 $$\begin{aligned} \Phi _{i0}'' - c_0 \Phi _{i0}' - \gamma _{\textrm{K}} \left( \Phi _{i0} - \phi _{\textrm{K}} \right) = 0. \end{aligned}$$

When $\Phi _{i0} \ge \Phi _{\textrm{th}}$, Eq. (24) becomes:

26 $$\begin{aligned} \Phi _{i0}'' - c_0 \Phi _{i0}' - \gamma _{\textrm{Na}} \left( \Phi _{i0} - \phi _{\textrm{Na}} \right) - \gamma _{\textrm{K}} \left( \Phi _{i0} - \phi _{\textrm{K}} \right) = 0. \end{aligned}$$

We required that $\Phi _{i0}$ and $\Phi _{i0}'$ are continuous at $\xi = \xi ^*$, with $\Phi _{i0}(\xi ^*) = \Phi _{\textrm{th}}$, where $\phi _{\textrm{Na}} = 1$ and $\phi _{\textrm{K}} = 0$. The solution should approach 0 as $\xi \rightarrow -\infty$ and $\frac{\gamma _{\textrm{Na}}}{\gamma _{\textrm{Na}} + \gamma _{\textrm{K}}}$ as $\xi \rightarrow \infty$. We therefore have:

$$\begin{aligned} \Phi _{i0}= &  D_1 e^{\lambda _1 \xi }, \quad \xi < \xi ^*\\ \Phi _{i0}= &  D_2 e^{\lambda _2 \xi } + \frac{\gamma _{\textrm{Na}}}{\gamma _{\textrm{Na}} + \gamma _{\textrm{K}}}, \quad \xi > \xi ^* \end{aligned}$$

where $\lambda _1 = \frac{c_0 + \sqrt{c_0^2 + 4 \gamma _{\textrm{K}}}}{2} > 0$ and $\lambda _2 = \frac{c_0 - \sqrt{c_0^2 + 4 (\gamma _{\textrm{Na}} + \gamma _{\textrm{K}})}}{2} < 0$. By continuity, we obtained the following equations:

27 $$\begin{aligned} \begin{aligned}&D_1 e^{\lambda _1 \xi ^*} = \Phi _{\textrm{th}}, \\&D_2 e^{\lambda _2 \xi ^*} + \frac{\gamma _{\textrm{Na}}}{\gamma _{\textrm{Na}} + \gamma _{\textrm{K}}} = \Phi _{\textrm{th}}, \\&D_1 \lambda _1 e^{\lambda _1 \xi ^*} = D_2 \lambda _2 e^{\lambda _2 \xi ^*}. \end{aligned} \end{aligned}$$

We need to solve for $D_1$, $D_2$, and $c_0$. While $\xi ^*$ can be any value, we take $\xi ^* = 0$ for simplicity. This gives:

$$\begin{aligned} D_1 = \Phi _{\textrm{th}}, \quad D_2 = \Phi _{\textrm{th}} - P_s = \Phi _{\textrm{th}} - \frac{\gamma _{\textrm{Na}}}{\gamma _{\textrm{Na}} + \gamma _{\textrm{K}}}, \quad c_0 = \frac{D}{\sqrt{4 \gamma _{\textrm{K}} + 2 D}}, \end{aligned}$$

where $$ D = 2 \left( (\gamma _{\textrm{Na}} + \gamma _{\textrm{K}}) \left( \frac{P_s}{\Phi _{\textrm{th}}} - 2 \right) + \frac{\gamma _{\textrm{Na}} \Phi _{\textrm{th}}}{P_s} \right) . $$

For the linear term in $\alpha$, we obtained:

28 $$\begin{aligned} \begin{aligned}&\Phi _{i1}''-c_0\Phi _{i1}'+f_{\textrm{side}}'(\Phi _{i0})\Phi _{i1}=c_1\Phi _{i0}'+\sigma \Phi _{c1},\\&2c_0(\Phi _{i0}-\Phi _{c0})'=2f_{\textrm{end}}(\Phi _{i0}-\Phi _{c0})+\sigma \Phi _{c1} \end{aligned} \end{aligned}$$

From Eq. (28), we infered the following:

29 $$\begin{aligned} \Phi _{i1}''-c_0\Phi _{i1}'+f'_{\textrm{side}}(\Phi _{i0})\Phi _{i1}=2c_0(\Phi _{i0}-\Phi _{c0})'-2f_{\textrm{end}}(\Phi _{i0}-\Phi _{c0})+c_1\Phi _{i0}'\nonumber \\ \end{aligned}$$

Equation (29) is a second-order linear differential equation with constant coefficients. We computed $c_1$ as follows. Let

$$\begin{aligned}Lu=u''-c_0u'+f'_{\textrm{side}}(\Phi _{i0})u,\end{aligned}$$

then $L[\cdot ]$ is a linear differential operator acting on *u*. Eq. (29) can be written as

$$\begin{aligned}L(\Phi _{i1})=2c_0(\Phi _{i0}-\Phi _{c0})'-2f_{\textrm{end}}(\Phi _{i0}-\Phi _{c0})+c_1\Phi _{i0}'.\end{aligned}$$

Since $L(\Phi _{i0}') = 0$ (which follows from taking derivatives of Eq. (24) with respect to $\xi$), we deduced that $L$ is not an invertible operator. According to the Fredholm alternative theorem, there is a solution to Eq. (29) if and only if the right-hand side is orthogonal to the null space of the adjoint operator $L^*$, where $L^* u = u'' + c_0 u' + f_{\textrm{side}}'(\Phi _{i0}) u$. Given that $L(\Phi _{i0}') = 0$, we claimed that $\Phi _{i0}' e^{-c_0 \xi }$ is the only element in the null space of $L^*$. This leads to the following condition for solvability:

$$\begin{aligned} \begin{aligned}&\langle 2c_0 (\Phi _{i0} - \Phi _{c0})' - 2 f_{\textrm{end}} (\Phi _{i0} - \Phi _{c0}) + c_1 \Phi _{i0}', \Phi _{i0}' e^{-c_0 \xi } \rangle = 0, \end{aligned} \end{aligned}$$

which results in the expression for $c_1$:

$$\begin{aligned} c_1 = -2c_0 + 2 \frac{\langle f_{\textrm{end}}(\Phi _{i0}), \Phi _{i0}' e^{-c_0 \xi } \rangle }{\langle \Phi _{i0}', \Phi _{i0}' e^{-c_0 \xi } \rangle }. \end{aligned}$$

To determine the sign of $c_1$, we computed its explicit expression as follows:

30 $$\begin{aligned} c_1= &  -2c_0 + 2 \frac{\langle f_{\textrm{end}}(\Phi _{i0}), \Phi _{i0}' e^{-c_0 \xi } \rangle }{\langle \Phi _{i0}', \Phi _{i0}' e^{-c_0 \xi } \rangle } \nonumber \\= &  -2c_0 + 2 \frac{\int _{-\infty }^{+\infty } f_{\textrm{end}}(\Phi _{i0}) \Phi _{i0}' e^{-c_0 \xi } d\xi }{\int _{-\infty }^{+\infty } \Phi _{i0}'^2 e^{-c_0 \xi } d\xi } \nonumber \\= &  -2c_0 + 2 \frac{\int _{-\infty }^0 f_{\textrm{end}}(\Phi _{i0}) \Phi _{i0}' e^{-c_0 \xi } d\xi + \int _0^{+\infty } f_{\textrm{end}}(\Phi _{i0}) \Phi _{i0}' e^{-c_0 \xi } d\xi }{\int _{-\infty }^0 \Phi _{i0}'^2 e^{-c_0 \xi } d\xi + \int _0^{+\infty } \Phi _{i0}'^2 e^{-c_0 \xi } d\xi } \nonumber \\= &  -2c_0 + 2 \frac{D_1 \lambda _1 \int _{-\infty }^0 f_{\textrm{end}}(\Phi _{i0}) e^{(\lambda _1 - c_0)\xi } d\xi + D_2 \lambda _2 \int _0^{+\infty } f_{\textrm{end}}(\Phi _{i0}) e^{(\lambda _2 - c_0)\xi } d\xi }{D_1^2 \lambda _1^2 \int _{-\infty }^0 e^{(2\lambda _1 - c_0)\xi } d\xi + D_2^2 \lambda _2^2 \int _0^{+\infty } e^{(2\lambda _2 - c_0)\xi } d\xi } \nonumber \\= &  -2c_0 + 2 \frac{D_1 \lambda _1 \left( \int _{-\infty }^{-\frac{\ln \beta }{\lambda _1}} f_{\textrm{end}}(\Phi _{i0}) e^{(\lambda _1 - c_0)\xi } d\xi + \int _{-\frac{\ln \beta }{\lambda _1}}^0 f_{\textrm{end}}(\Phi _{i0}) e^{(\lambda _1 - c_0)\xi } d\xi \right) }{\frac{D_1^2 \lambda _1^2}{s_1} + \frac{D_2^2 \lambda _2^2}{s_2}} \nonumber \\ &  +2\frac{D_2 \lambda _2 \int _0^{+\infty } f_{\textrm{end}}(\Phi _{i0}) e^{(\lambda _2 - c_0)\xi } d\xi }{\frac{D_1^2 \lambda _1^2}{s_1} + \frac{D_2^2 \lambda _2^2}{s_2}} \nonumber \\= &  -2c_0 + 2 \frac{\text {top1} + \text {top2}}{\frac{D_1^2 \lambda _1^2}{s_1} + \frac{D_2^2 \lambda _2^2}{s_2}}, \end{aligned}$$

where

$$\begin{aligned} s_1= &  \sqrt{c_0^2 + 4 \gamma _{\textrm{K}}}, \quad s_2 = \sqrt{c_0^2 + 4 (\gamma _{\textrm{Na}} + \gamma _{\textrm{K}})},\\ \text {top1}= &  D_1 \lambda _1 \left( -\frac{\beta \gamma _{\textrm{Na}} \Phi _{\textrm{th}}}{s_1} + \frac{\beta ^{\frac{\lambda _1 - s_1}{\lambda _1}} \gamma _{\textrm{Na}} \Phi _{\textrm{th}}}{s_1} - \frac{\gamma _{\textrm{K}} \Phi _{\textrm{th}}}{s_1} + \frac{\beta \gamma _{\textrm{Na}}}{\lambda _1 - c_0} - \frac{\beta ^{\frac{c_0}{\lambda _1}} \gamma _{\textrm{Na}}}{\lambda _1 - c_0} \right) , \end{aligned}$$

and

$$\begin{aligned} \text {top2} = -\frac{D_2^2 \lambda _2 (\beta \gamma _{\textrm{Na}} + \gamma _{\textrm{K}})}{s_2} + \frac{2 D_2 \lambda _2 \left( (1 - P_s) \beta \gamma _{\textrm{Na}} - P_s \gamma _{\textrm{K}} \right) }{c_0 + s_2}. \end{aligned}$$

**Fig. 1:**
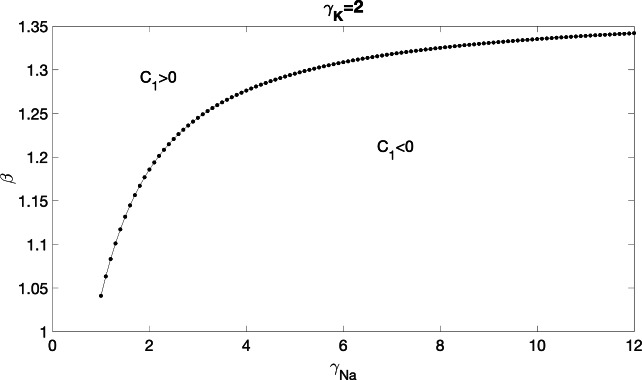
Curve $c_1=0$ and regions $c_1>0$, $c_1<0$ in the $\gamma _{\textrm{Na}}$–$\beta$ plane with $\gamma _{\textrm{K}}=2$.

From Eq. (30), it is clear that the expression of $c_1$ is determined by $\Phi _{\textrm{th}}$, $\gamma _{\textrm{Na}}$, $\gamma _{\textrm{K}}$, and $\beta$. However, since $\Phi _{\textrm{th}}$ appears in combination with $\beta$ when describing the activation of the end membrane, we only considered the impact of $\gamma _{\textrm{Na}}$, $\gamma _{\textrm{K}}$, and $\beta$ on $c_1$. To fully understand the factors controlling the sign of $c_1$, we plotted the curve $c_1=0$ together with the regions where $c_1>0$ and $c_1<0$ for $\gamma _{\textrm{Na}}$–$\beta$ combinations with $\gamma _{\textrm{K}}=2$ (Fig. 1). We found that $\beta$ plays the dominant role in determining the sign of $c_1$, and that an increase in $\gamma _{\textrm{Na}}$ shifts the threshold $\beta$ required for $c_1>0$ upward, implying that weak EpC can also increase CV when the $I_{\textrm{Na}}$ channels are localized. This finding is consistent with experimental observations showing $I_{\textrm{Na}}$ channel localization to the cleft (Rhett et al. 2012). In contrast, variations in $\gamma _{\textrm{K}}$ and $\beta$ have little effect on the sign of $c_1$ (data not shown).

#### Cubic ionic model

Furthermore, we calculated the CV using the continuous model described in Eqs. (15)–(16), combined with the cubic ionic model outlined in Eq. (8). When $\alpha =0$, the solution is given by $\Phi _{i0}(\xi )=\frac{1}{2}\big (1+\textrm{tanh}(\frac{A\xi }{2\sqrt{2}})\big )$ and $c_0=\frac{A}{\sqrt{2}}(1-2\epsilon _{\textrm{side}})$ (Keener and Sneyd 2009). Using a perturbation argument for CV with small $\alpha$, we obtained

31 $$\begin{aligned} \begin{aligned} c_1&=-2c_0+2\frac{\langle f_{\textrm{end}}(\Phi _{i0}), \Phi _{i0}'e^{-c_0\xi } \rangle }{\langle \Phi _{i0}', \Phi _{i0}'e^{-c_0\xi }\rangle }\\&=-2c_0+2\frac{\int _{-\infty }^{+\infty }f_{\textrm{end}}(\Phi _{i0})\Phi _{i0}'e^{-c_0\xi }d\xi }{\int _{-\infty }^{+\infty }\Phi _{i0}'^2e^{-c_0\xi }d\xi }\\&=-2c_0+\frac{\sqrt{2}A\beta \int _{-\infty }^{+\infty }\textrm{sech}^4(\frac{A\xi }{2\sqrt{2}})\big (\tanh (\frac{A\xi }{2\sqrt{2}})+1-2\epsilon _{\textrm{end}}\big )e^{-c_0\xi }d\xi }{\int _{-\infty }^{+\infty }\textrm{sech}^4(\frac{A\xi }{2\sqrt{2}})e^{-c_0\xi }d\xi },\\ \end{aligned} \end{aligned}$$

#### Numerical validation for continuous model

In this section, we validated the analytical expression for the CV of the continuous model presented in Sec. 3.1 through numerical simulations. We solved Eqs. (15) and (16), combined with the piecewise linear and cubic ionic models described in Eqs. (7) and (8), respectively. The backward Euler method was used for time discretization, with Newton’s method applied to solve the resulting nonlinear equations, while the central difference method was employed for spatial discretization. The time step was set to $d\tau =0.0001$ and the spatial step to $dx=0.01$. The iteration in Newton’s method is terminated when the absolute value of the residual is less than $10^{-5}$, and the method exhibits quadratic convergence.

**Fig. 2:**
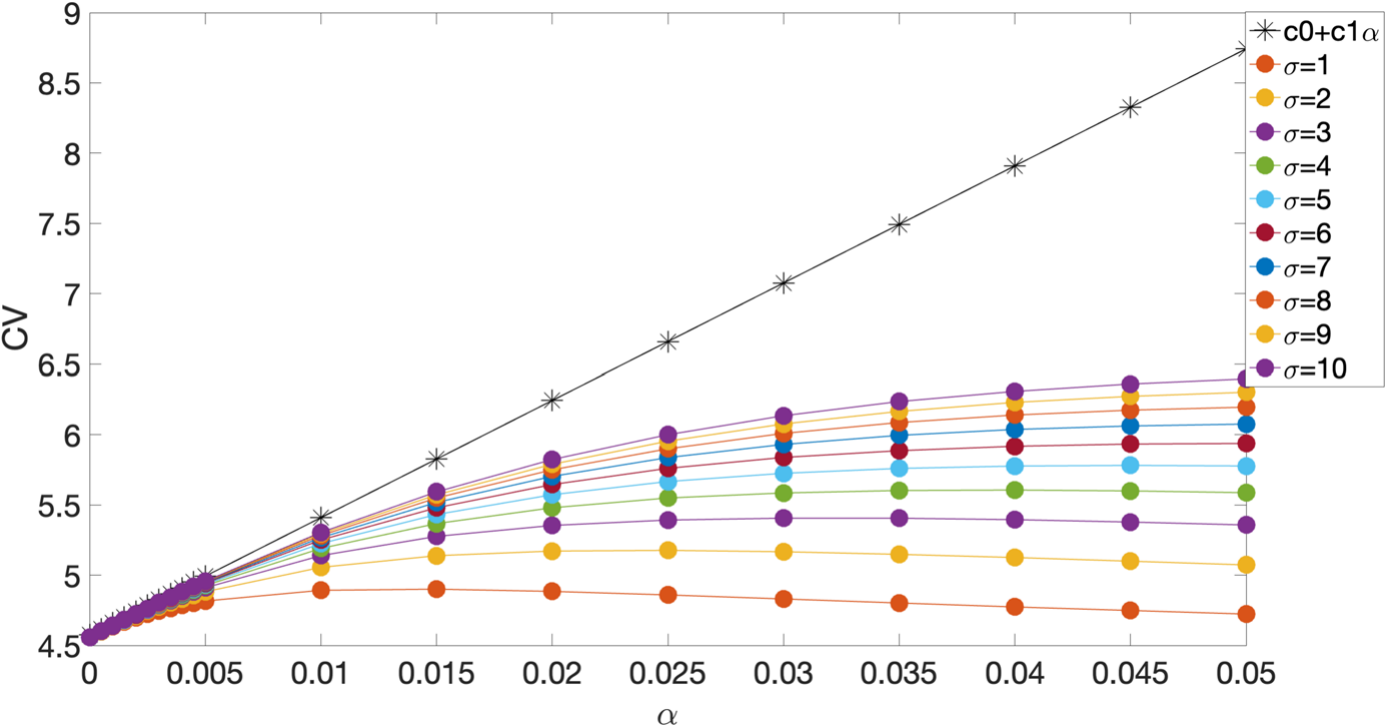
CV for both analytical (black stars) and numerical (filled circles) studies of the continuous model with piecewise linear ionic dynamics, as a function of $\alpha$, at different values of $\sigma$, with $\gamma _{\textrm{Na}}=6$, $\gamma _{\textrm{K}}=2$, $\beta =5$ and $\Phi _{\textrm{th}}=0.16$ (Copene and Keener 2008)

Fig. 2 compares the CV obtained from both analytical (black stars) and numerical (filled circles) methods as a function of $\alpha$ for various values of $\sigma$, where $\gamma _{\textrm{Na}}=6$, $\gamma _{\textrm{K}}=2$, $\beta =5$ and $\Phi _{\textrm{th}}=0.16$. As shown in the figure, the CV from the perturbation method is tangent to the CV curves from the numerical simulations close to $\alpha =0$. Furthermore, when $\alpha$ is sufficiently small ($\alpha \le 0.01$), the effect of $\sigma$ on CV is minimal, consistent with Eq. (30), in which $\sigma$ is not directly involved.

**Fig. 3:**
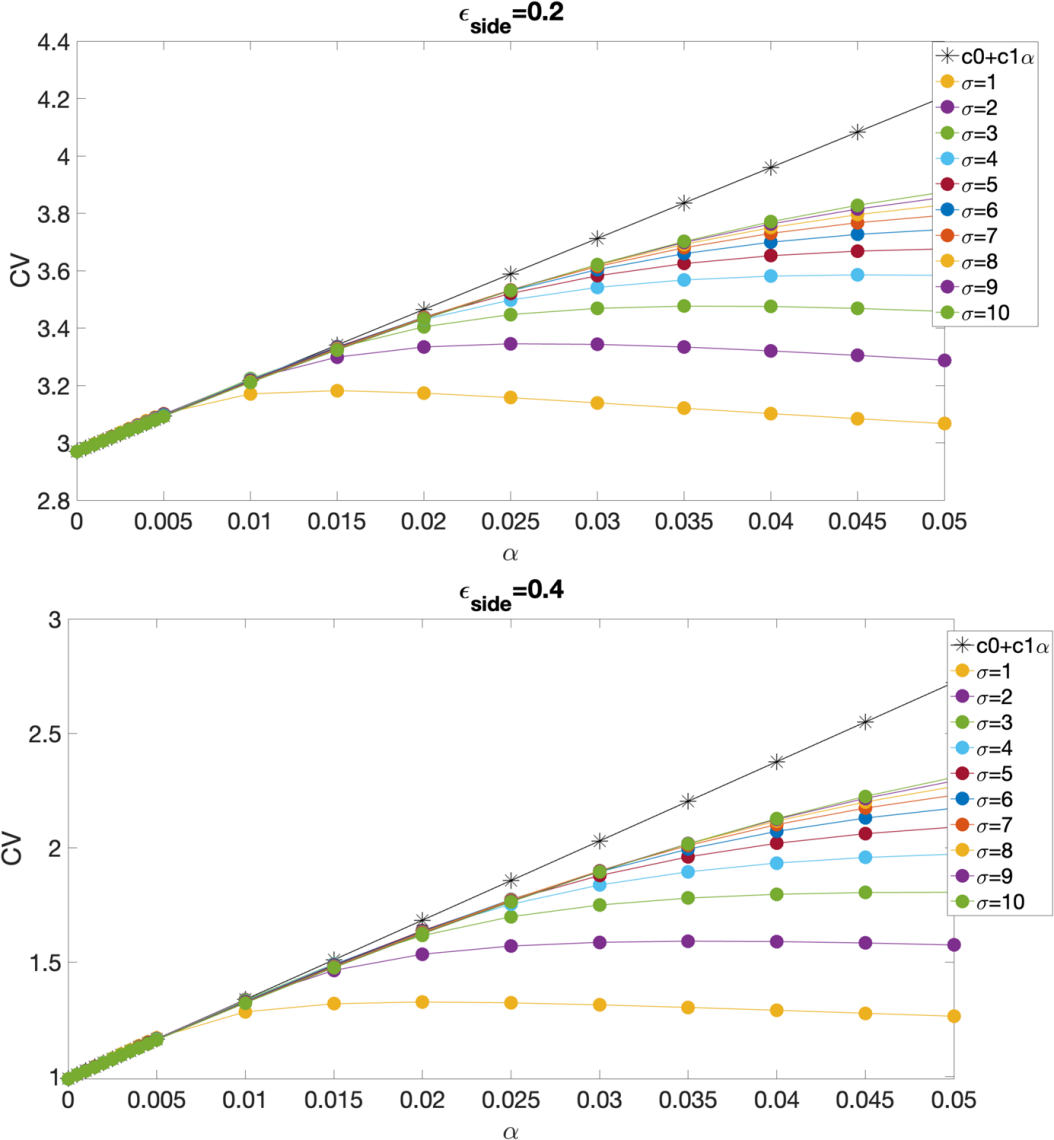
CV for both analytical (black stars) and numerical (filled circles) studies of the continuous model with cubic ionic dynamics, as a function of $\alpha$, at different values of $\sigma$, with $A=7$, $\epsilon _{\textrm{side}}=0.2$ (top) and 0.4 (bottom), $\beta =5$, $\epsilon _{\textrm{end}}=\frac{\epsilon _{\textrm{side}}}{\beta }$.

Fig. 3 compares the CV obtained using both analytical (black stars) and numerical (filled circles) methods as a function of $\alpha$ for different $\sigma$, where parameters used in the simulations were $A=7$, $\epsilon _{\textrm{side}}=0.2$ (top) and 0.4 (bottom), $\beta =5$, $\epsilon _{\textrm{end}}=\frac{\epsilon _{\textrm{side}}}{\beta }$ and $\Phi _{\textrm{th}}=0.5$. Similarly to Fig. 2, the CV derived from the perturbation method closely aligns with the CV curves from numerical simulations near $\alpha =0$. Additionally, when $\alpha$ is sufficiently small ($\alpha <=0.015$), the influence of $\sigma$ on CV becomes negligible.

### Conduction speed of discrete model when $\alpha$ is small

#### Formula derivation of the discrete model

In this section, we analyzed the discrete model given by Eqs. (5) and (6) to compute the CV of a traveling wave. For a traveling wave to exist, the following conditions must be satisfied:

$$\begin{aligned} \phi _i^{m-1}(\tau )= &  \phi _i(\tau -\tau _d), \phi _i^{m+1}(\tau )=\phi _i(\tau +\tau _d),\\ \phi _c^{m+\frac{1}{2}}(\tau )= &  \phi _c(\tau +\tau _d), \end{aligned}$$

where $\phi _i=\phi _i^m$ and $\phi _c=\phi _c^{m-\frac{1}{2}}$. In other words, the $(m-1)^{\textrm{th}}$ and $(m+1)^{\textrm{th}}$ nodes experience the same time course as the $m^{\textrm{th}}$ node, but with a time delay of $\tau _d$; similarly, the $(m+\frac{1}{2})^{\textrm{th}}$ node has the same time course as $(m-\frac{1}{2})^{\textrm{th}}$ node, with a time delay of $\tau _d$. Assuming that the functions $\phi _i$ and $\phi _c$ are sufficiently smooth and that $\tau _d$ is sufficiently small, we can expand these functions in Taylor series around $\tau$:

$$\begin{aligned} \phi _i(\tau -\tau _d)= &  \Sigma _{n=0}\frac{1}{n!}\phi _i^{(n)}(\tau )(-\tau _d)^n,\\ \phi _i(\tau +\tau _d)= &  \Sigma _{n=0}\frac{1}{n!}\phi _i^{(n)}(\tau )\tau _d^n,\\ \phi _c(\tau +\tau _d)= &  \Sigma _{n=0}\frac{1}{n!}\phi _c^{(n)}(\tau )\tau _d^n. \end{aligned}$$

For the discrete model, two parameters, $\alpha$ ($\ll$ 1) and $\tau _d$ ($\ll$ 1), both influence the speed of the action potential. We assumed that $\tau _d \sim \alpha$. Furthermore, to maintain the diffusion terms, we also imposed the condition that $\gamma _s \tau _d^2=O(1)$ as $\tau _d \rightarrow 0$. The equations (5) and (6) can be approximated by the following forms:

32 $$\begin{aligned} \begin{aligned}&\phi _i'+\alpha (\phi _i-\phi _c)'+\alpha \Big (\phi _i-\phi _c-{\phi _c}'\tau _d\Big )'\\&=\gamma _s({\phi _i}''\tau _d^2+\frac{\phi _i^{(4)}}{12}\tau _d^4)+f_{\textrm{side}}(\phi _i)\\&+\alpha f_{\textrm{end}}(\phi _i-\phi _c)+\alpha f_{\textrm{end}}\Big (\phi _i-\phi _c-\phi _c'\tau _d\Big ) \end{aligned} \end{aligned}$$

33 $$\begin{aligned} \alpha \Big (\phi _i-\phi _i'\tau _d-\phi _c\Big )' +\alpha (\phi _i-\phi _c)'=\alpha f_{\textrm{end}}(\phi _i-\phi _i'\tau _d-\phi _c)+\alpha f_{\textrm{end}}(\phi _i-\phi _c) +\sigma \phi _c \nonumber \\ \end{aligned}$$

The leading order equations are the following:

34 $$\begin{aligned} \begin{aligned}&\phi _i'+2\alpha (\phi _i-\phi _c)'=\gamma _s\tau _d^2\phi _i''+f_{\textrm{side}}(\phi _i)+2\alpha f_{\textrm{end}}(\phi _i-\phi _c) \end{aligned} \end{aligned}$$

35 $$\begin{aligned} \begin{aligned}&2\alpha (\phi _i-\phi _c)'=2\alpha f_{\textrm{end}}(\phi _i-\phi _c) +\sigma \phi _c\\ \end{aligned} \end{aligned}$$

These equations can be rewritten as follows:

36 $$\begin{aligned} \begin{aligned}&\gamma _s\tau _d^2\phi _i''-\phi _i'+f_{\textrm{side}}(\phi _i)=\sigma \phi _c \end{aligned} \end{aligned}$$

37 $$\begin{aligned} \begin{aligned}&(\phi _i-\phi _c)'= f_{\textrm{end}}(\phi _i-\phi _c) +\frac{\sigma }{2\alpha } \phi _c\\ \end{aligned} \end{aligned}$$

We assumed that Eqs. (36) and (37) have solutions

$$\begin{aligned}\phi _{i0}(\tau )=\Phi _i(c\tau ), \phi _{c0}(\tau )=\Phi _c(c\tau ),\end{aligned}$$

where $\Phi _i$ and $\Phi _c$ are the traveling wave solutions of the following continuous equations, with $\gamma _s\tau _d^2=1/c^2$, and $c(=c_0+c_1\alpha )$ representing the dimensionless wave speed of the following model.

38 $$\begin{aligned} \begin{aligned}&\Phi _i^{''}-c\Phi _i^{'}+f_{\textrm{side}}(\Phi _i)=\sigma \Phi _c,\\&c(\Phi _i'-\Phi _c')=f_{\textrm{end}}(\Phi _i-\Phi _c)+\frac{\sigma }{2\alpha }\Phi _c. \end{aligned} \end{aligned}$$

The wave speed *v* of the discrete model is then given by

$$\begin{aligned}v=\frac{\Delta x}{\tau _d}=c\Delta x\sqrt{\gamma _s}=c\sqrt{\Delta x^2\gamma _s}=c\sqrt{\gamma ^*}, \text {where}\;c=c_0+c_1\alpha .\end{aligned}$$

The derived formula for the CV in the discrete models applies to both piecewise linear and cubic ionic dynamics.

#### Numerical validation of the discrete model

In this section, we validated the analytical expression for the CV of the discrete model presented through numerical simulations. We solved Eqs. (5) and (6), combined with the piecewise linear and cubic ionic models described in Eqs. (7) and (8), respectively. The backward Euler method was used for time discretization, with newton’s method applied to solve the resulting nonlinear equations, using a time step of $d\tau = 0.0001$. Similar to the continuous model, Newton’s method is terminated when the absolute value of the residual is less than $10^{-5}$ and has quadratic convergence.

**Fig. 4:**
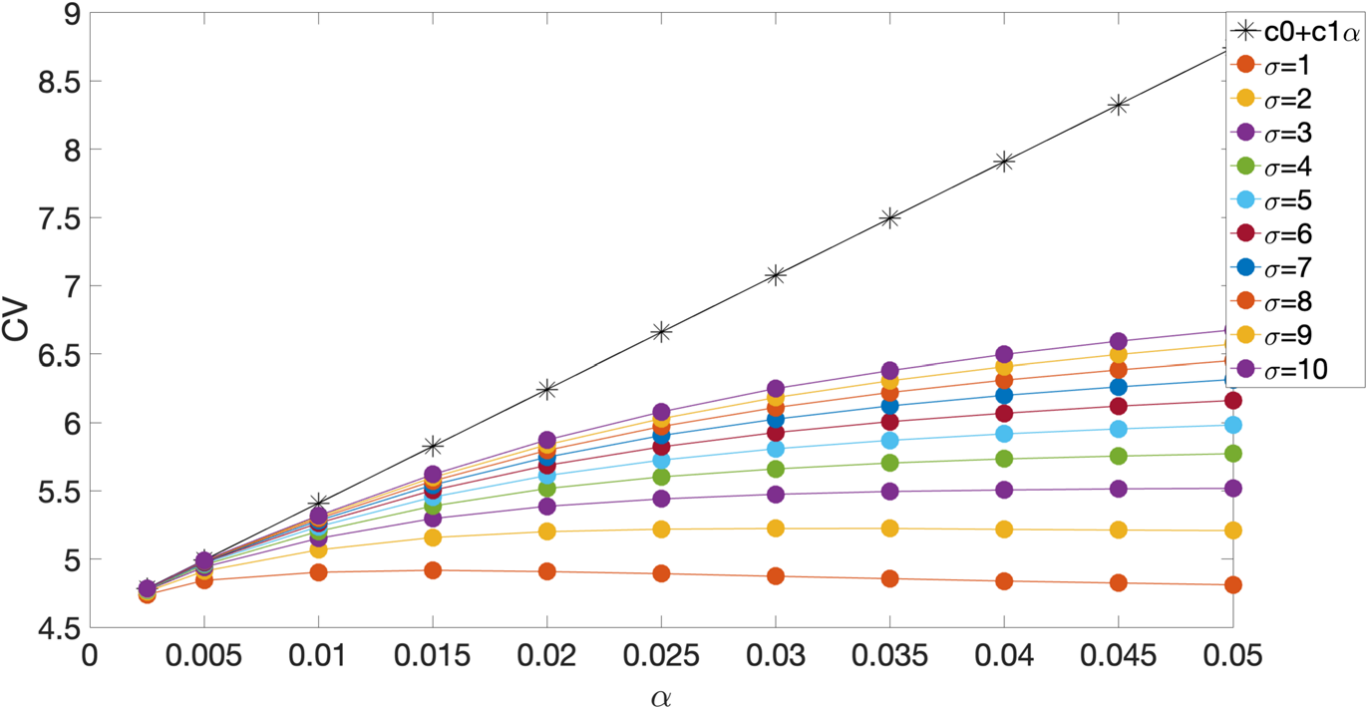
CVs for both analytical ($s > \frac{1}{2}$, black stars) and numerical (filled circles) studies of the discrete model with piecewise linear ionic dynamics, as a function of $\alpha$, at different values of $\sigma$, with $\gamma _{\textrm{Na}} = 6$, $\gamma _{\textrm{K}} = 2$, $\beta = 5$, and $\Phi _{\textrm{th}} = 0.16$.

We first validated our analytical expression for the CV for the piecewise linear ionic models. Fig. 4 compares the CVs obtained from both the analytical solution and numerical methods as a function of $\alpha$ for various values of $\sigma$, with $\gamma ^* = 1$, consistent with the value used in the continuous model. The parameters we used are $\gamma _{\textrm{Na}} = 6$, $\gamma _{\textrm{K}} = 2$, $\beta = 5$, and $\Phi _{\textrm{th}} = 0.16$. As shown in the figure, the CV from the analytical solution is tangent to the numerical CV curves close to $\alpha = 0$. Moreover, for sufficiently small values of $\alpha$ ($\alpha \le 0.01$), the impact of $\sigma$ on the CV is negligible, which aligns with the result from the continuous model.

**Fig. 5:**
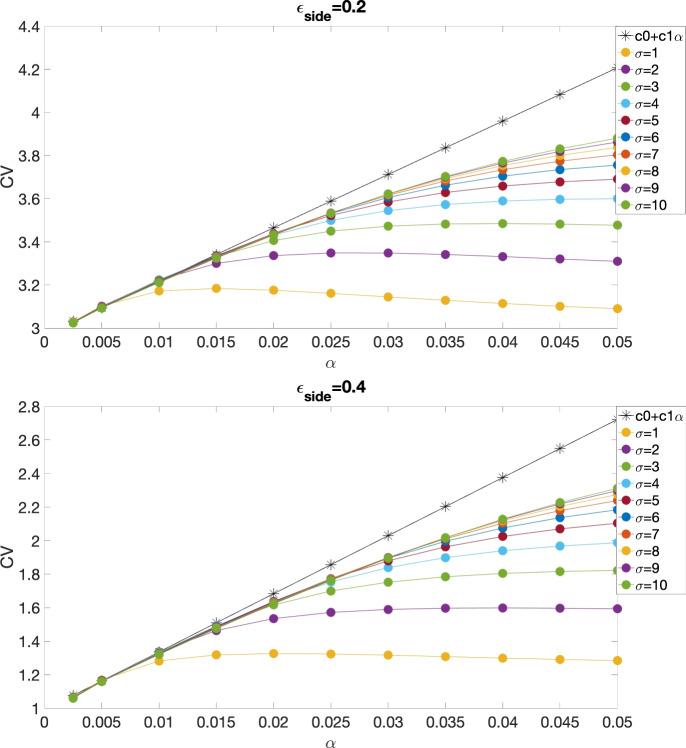
CV for both analytical (black stars) and numerical (filled circles) studies of the discrete model with cubic ionic dynamics, as a function of $\alpha$, at different values of $\sigma$, with $A=7$, $\epsilon _{\textrm{side}}=0.2$ (top) and 0.4 (bottom), $\beta =5$, $\epsilon _{\textrm{end}}=\frac{\epsilon _{\textrm{side}}}{\beta }$.

We also validated our analytical expression for the CV in the cubic ionic models. Fig. 5 compares the CVs obtained from both the analytical solution and numerical methods as a function of $\alpha$ for various values of $\sigma$, with $\gamma ^* = 1$, consistent with the value used in the continuous model. The parameters used are $A = 7$, $\beta = 5$, $\epsilon _{\textrm{side}} = 0.2$ (top) and 0.4 (bottom), and $\epsilon _{\textrm{end}} = \frac{\epsilon _{\textrm{side}}}{\beta }$. As shown in the figure, the CV from the analytical solution, represented by the line $c_0 + c_1 \alpha$, is tangent to the numerical CV curves near $\alpha = 0$, indicating good agreement between the analytical and numerical results.

## Discussion

The CV for ephaptic conduction has been numerically studied over the past few decades across various levels of GJs (Kucera et al. 2002; Hand et al. 2009; Hand and Peskin 2010; Lin and Keener 2014; Mori et al. 2008; Veeraraghavan et al. 2014; Copene and Keener 2008; Picone et al. 1991; Lin and Keener 2013; Wei et al. 2016; Wei and Tolkacheva 2020; George et al. 2015). When GJs are strong, CV increases monotonically and nonlinearly as $d_{\textrm{cleft}}$ increases (Wei et al. 2016; Hand et al. 2009; Hand and Griffith 2010; Hand and Peskin 2010; Kucera et al. 2002); that is, strong EpC can reduce CV nonlinearly. However, when GJs are weak, EpC can increase CV (Wei et al. 2016; Hand et al. 2009; Hand and Griffith 2010; Hand and Peskin 2010; Kucera et al. 2002). In particular, CV exhibits a biphasic relationship with respect to $d_{\textrm{cleft}}$, with a sufficiently small optimal $d_{\textrm{cleft}}$ at which CV is maximal. However, most of these studies are either numerical or experimental in nature. The only analytical study of ephaptic conduction (Copene and Keener 2008) examines its feasibility in the absence of GJs. This study describes two cells coupled through a shared junctional cleft potential, where successful conduction is highly dependent on specific parameters. The authors categorized the parameter space into regions of propagation success and two distinct types of propagation failure. The analytical expression for the CV of ephaptic conduction, though equally important for understanding EpC, has not been thoroughly explored.

In our study, we employed both continuous and discrete models, which are coupled with a bistable ionic equation (piecewise linear and cubic). Although the discrete model might seem like a finite difference approximation of the continuous model, it is actually more complex than the continuous model. In the discrete model, we account for two parameters, $\tau _d$ and $\alpha$, which represent the discreteness and ephaptic effects of the model, respectively. Both parameters influence the CV. We assumed that $\tau _d \sim \alpha ^s$ and explored three different values for the exponent *s* ($s>0$): $s < \frac{1}{2}$, $s = \frac{1}{2}$, and $s > \frac{1}{2}$. Furthermore, to maintain the diffusion terms, we also imposed the condition that $\gamma _s \tau _d^2=O(1)$ as $\tau _d \rightarrow 0$. For the case where $0<s<\frac{1}{2}$, the leading-order equation derived from Eqs. (32) and (33) is the following:

39 $$\begin{aligned} \phi _i'=\gamma _s(\phi _i''\tau _d^2+\frac{\phi _i^{(4)}}{12}\tau _d^4)+f_{\textrm{side}}(\phi _i). \end{aligned}$$

Here, EpC has been omitted. Therefore, this does not represent the desired form. Next, we analyzed the case where $s>\frac{1}{2}$, in which the leading-order equations are identical to those for $s=1$ as presented in the results section. Finally, we examined the case where $s=\frac{1}{2}$, that is $\tau _d\sim \alpha ^{\frac{1}{2}}$. The leading order equations derived from Eqs. (32) and (33) are as follows:

40 $$\begin{aligned} \phi _i'+2\alpha (\phi _i-\phi _c)'= &  \gamma _s(\tau _d^2{\phi _i}''+\frac{\tau _d^4}{12}\phi _i^{(4)})+f_{\textrm{side}}(\phi _i)+2\alpha f_{\textrm{end}}(\phi _i-\phi _c)\end{aligned}$$

41 $$\begin{aligned} 2\alpha (\phi _i-\phi _c)'= &  2\alpha f_{\textrm{end}}(\phi _i-\phi _c) +\sigma \phi _c. \end{aligned}$$

From Eqs. (40) and (41), it is clear that both EpC and the higher order approximations play an equivalent role. In particular, as $\alpha \rightarrow$ 0, both EpC and the higher order approximation vanish simultaneously. In the leading order equations, $\phi _{i}^{(4)}$ appears, indicating that the formulation of $\phi _{i}$ corresponding to the piecewise linear ionic model is not appropriate for computing CV. As a result, we opted to replace it with the cubic ionic model described in Eq. (8). The analytical expression is $v^*=c^*\sqrt{\gamma ^*},$ where $c^*=c_0^*+c_1^*\alpha$. $c_0^*=c_0$ and

42 $$\begin{aligned} \begin{aligned} c_1^*&=-\frac{\langle -\frac{1}{12}c_0^{*2}\Phi _{i0}^{(4)}+2c_0^*\Phi _{i0}'-2f_{\textrm{end}}(\Phi _{i0}),\Phi _{i0}'e^{-c_0^*\xi }\rangle }{\langle \Phi _{i0}^{'},\Phi _{i0}'e^{-c_0^*\xi }\rangle }\\&=-\frac{\langle -\frac{1}{12}c_0^{2}\Phi _{i0}^{(4)},\Phi _{i0}'e^{-c_0\xi }\rangle }{\langle \Phi _{i0}^{'},\Phi _{i0}'e^{-c_0\xi }\rangle }-2c_0+\frac{\langle 2f_{\textrm{end}}(\Phi _{i0}),\Phi _{i0}'e^{-c_0\xi }\rangle }{\langle \Phi _{i0}^{'},\Phi _{i0}'e^{-c_0\xi }\rangle }\\&=\frac{\frac{1}{12}c_0^{2}\langle \Phi _{i0}^{(4)},\Phi _{i0}'e^{-c_0\xi }\rangle }{\langle \Phi _{i0}^{'},\Phi _{i0}'e^{-c_0\xi }\rangle }+c_1\\&=\frac{\frac{A^3c_0^2}{24\sqrt{2}}\int _{-\infty }^{+\infty }\tanh (\frac{A\xi }{2\sqrt{2}})\textrm{sech}^4(\frac{A\xi }{2\sqrt{2}})\left( 2\textrm{sech}^2(\frac{A\xi }{2\sqrt{2}})-\tanh ^2(\frac{A\xi }{2\sqrt{2}})\right) e^{-c_0\xi }}{\int _{-\infty }^{+\infty }\textrm{sech}^4(\frac{A\xi }{2\sqrt{2}})e^{-c_0\xi }d\xi }+c_1. \end{aligned} \end{aligned}$$

Here $c_0$ and $c_1$ denote the leading-order and first-order approximations of the CV for $s>\frac{1}{2}$. We also compared the analytical expression with numerical simulations and found good agreement when $\epsilon _{\textrm{side}}$ is close to 0.5. However, some inconsistencies arise when $\epsilon _{\textrm{side}}$ deviates significantly from 0.5. Addressing this discrepancy will be part of future work.

One limitation of our study is that we used the asymptotic series for small $\alpha$, which represents weak EpC. It is a future challenge to develop a reasonable analytically tractable model in the case of strong EpC. Another limitation of our study is the use of simplified piecewise linear and cubic ionic models. However, these models are sufficient to capture the depolarization phase of the action potential and are essential for enabling mathematical analysis.

## Conclusion

In this paper, we applied asymptotic theory to calculate the CV in the presence of weak EpC. To achieve this, we developed both continuous and discrete models to describe ephaptic conduction along a strand of cells. Ionic dynamics were modeled using the piecewise linear and cubic functions. The resulting system represents a bistable system with weak EpC. We calculated an expression for CV in the presence of weak EpC for both models, and validated our analytical results with numerical simulations. Additionally, we showed that under weak EpC, CV can increase if the distribution of INa is more prominent on the end membrane (i.e., when $\beta \geqslant 1.4$).

## Data Availability

The data for this paper is already included in the manuscript.
